# What makes embedded implementation research work? A multicountry synthesis of UNICEF-supported initiatives in low- and middle-income countries

**DOI:** 10.7189/jogh.16.04265

**Published:** 2026-07-31

**Authors:** ASM Shahabuddin, Robert Scherpbier, Alyssa Sharkey, Muzhgan Haydary, Kumanan Rasanathan, Ann Robins, Karin Källander

**Affiliations:** 1 Maternal, Newborn, Child and Adolescent Health Section, Health Programme Group, UNICEF, New York, USA; 2 School of Public and International Affairs, Princeton University, Princeton, New Jersey, USA; 3 Centre for Public Health Research, University of Montreal, Montreal, Quebec, Canada; 4 Department of Global Public Health, Karolinska Institutet, Stockholm, Sweden; 5 Alliance for Health Policy and Systems Research, World Health Organization, Geneva, Switzerland

**Keywords:** embedded implementation research, health systems, policy uptake, service delivery, UNICEF, LMICs

## Abstract

**Introduction:**

Embedded implementation research (EIR) integrates research within health programmes to address delivery bottlenecks and improve uptake of evidence. While widely promoted, its influence on policy, programme delivery, and service outcomes in low- and middle-income countries (LMICs) remains under-documented. We examined how EIR has influenced policy adaptation, programme design, and service delivery outcomes across LMICs where UNICEF has supported EIR initiatives.

**Methods:**

We conducted a mixed-methods cross-sectional synthesis drawing on survey data, document review, and case analyses of UNICEF-supported EIR projects conducted between 2015 and 2022. The analysis was guided by Proctor’s implementation outcomes framework (adaptation, fidelity, sustainability) and the Consolidated Framework for Implementation Research to explore how contextual mechanisms shaped observed outcomes. We included 33 researchers and implementers representing 24 completed EIR projects from 11 countries who participated in an online structured survey (69% response rate). Their responses were triangulated with project reports and UNICEF monitoring documentation.

**Results:**

Two-thirds (67%) of projects reported that their findings informed policy or programme adjustments, most commonly through revised immunisation strategies, integration of digital tools, and strengthened community engagement. Half (50%) documented measurable service-delivery improvements, such as increased vaccination coverage and improved routine child health indicators, reflecting greater fidelity to evidence-informed practices. Examples from Pakistan, Malawi, and Ethiopia demonstrated policy adaptation and scaling of interventions derived from EIR evidence. Across projects, early engagement of decision-makers, alignment with programme cycles, and participatory dissemination were key enablers of uptake. Persistent barriers included limited political commitment, competing priorities, and inadequate post-research financing.

**Conclusions:**

When co-led by decision-makers, integrated into programme cycles and national coordination structures, embedded implementation research can accelerate the use of evidence and strengthen programme performance. To maximise its potential, future EIR efforts should prioritise sustained political commitment, dedicated financing, and mechanisms for ongoing follow-up and learning to ensure that research findings translate into policy and service-delivery improvements.

The persistent gap between research and policy uptake remains a major barrier to achieving health equity, particularly in low- and middle-income countries (LMICs) [1]. Embedded implementation research (EIR) has emerged as a promising approach to address this challenge by integrating research with ongoing health programmes and decision-making cycles [2,3]. Unlike conventional research models, EIR is co-led by programme managers and decision-makers and aims to generate context-specific evidence in real time to improve service delivery and health outcomes [4].

By addressing research questions of direct relevance to programmes, aligning research activities with implementation cycles, and carrying out research in collaboration with local partners, EIR can increase the likelihood of policies and programmes being evidence-based and correcting course in response to learning on challenges [3,5–7]. The local context-the social, political and economic environment in which the program operates-is a crucial factor considered in EIR [2,3,5,8].

Since 2015, UNICEF has supported over 100 EIR projects, defining the approach as ‘the integration of research within existing programme implementation and policymaking to improve outcomes and overcome implementation bottlenecks’ [4]. These projects are largely implemented within UNICEF’s Maternal, Newborn, Child and Adolescent Health (MNCAH) programme, which supports countries in strengthening health systems and improving equitable access to essential services across the life course. Embedding implementation research within MNCAH programmes provides an opportunity to generate context-specific evidence directly linked to programme delivery and policy priorities. These projects are designed to align research with implementation cycles, directly respond to bottlenecks identified by local stakeholders, and embed inquiry within health systems to ensure relevance, ownership, and rapid uptake [5–8].

UNICEF’s EIR portfolio spans over 25 countries, largely in partnership with Gavi, the United States Agency for International Development, the Gates Foundation, and Global Affairs Canada, and has targeted priority child health areas, including immunisation, maternal and newborn care, nutrition, and civil registration, in both development and emergency settings. This approach was co-developed with the Alliance for Health Policy and Systems Research at the World Health Organization (WHO) and implemented in collaboration with academic partners across the Global South [9–12].

While EIR has gained momentum globally, systematic assessments of its impact on programme adaptation, policy uptake, and service delivery outcomes remain limited. This study addresses this gap by synthesising data and insights from UNICEF-supported EIR projects across several LMICs. We examined how EIR influenced service delivery, programme design, and policy change and identified cross-cutting lessons that can inform future EIR practice and policy engagement strategies across LMIC settings.

## METHODS

### Study design and conceptual framework

We employed a mixed-methods cross-sectional study design to understand how EIR contributed to policy uptake, programme adaptation and service delivery improvements across LMICs. EIR was conceptualised as an approach aimed at institutionalising the use of evidence within health systems, rather than as a discrete research project. We analysed how the EIR approach itself was implemented, adopted and sustained and how it influenced policy and programme outcomes. Quantitative data from structured survey responses were used to describe the frequency and distribution of reported outcomes across projects. Qualitative data from open-ended responses and document review were analysed to explore how and under what conditions these outcomes occurred.

The analysis was guided by two complementary implementation science frameworks. We used Proctor’s taxonomy of implementation outcomes to assess key dimensions of programme and policy change, including adaptation, fidelity, and sustainability [13]. We applied the Consolidated Framework for Implementation Research (CFIR) to examine contextual factors influencing these outcomes, including intervention characteristics, inner and outer settings, individual actors, and implementation processes [14]. Together, these frameworks enabled a structured analysis of both reported service delivery outcomes and the contextual factors shaping their uptake. We focused on the perspectives of researchers and implementers directly engaged in EIR to understand mechanisms of influence in real-world settings. The inclusion of policymakers and quantitative impact assessments would provide complementary insights but were beyond the scope of this analysis.

### Study setting and participants

We drew on UNICEF’s global portfolio of EIR projects conducted between 2015 and 2022, in collaboration with national ministries of health, research institutions, and implementation partners.

From over 100 EIR projects, we purposively selected 40 completed projects based on the following criteria: completion of the EIR cycle, availability of documentation and dissemination outputs, and representation across geographic regions and thematic areas, including immunisation, maternal and child health, and nutrition. With this purposive sampling approach we aimed at capturing a diverse range of implementation contexts and experiences rather than to achieve statistical representativeness.

Participants were eligible if they had direct involvement in the design, implementation, or use of EIR findings and could provide informed perspectives on project processes and outcomes. We excluded participants with limited involvement or insufficient knowledge of the EIR project. We invited 48 participants to an online survey; 33 respondents, representing 24 projects across 11 countries, completed it (69% response rate). Non-response was primarily due to staff turnover, limited availability, or outdated contact information. While some projects are represented by a limited number of respondents, triangulation with project documentation and UNICEF monitoring data was used to enhance validity and reduce individual-level bias.

### Data collection

We collected data in the third quarter of 2023 using a semi-structured online questionnaire designed to capture both quantitative and qualitative information on EIR processes and outcomes. The questionnaire included closed-ended items to document the presence and types of reported programme, policy and service delivery changes, as well as open-ended questions to explore enabling factors, barriers, and sustainability of EIR initiatives.

Respondents were asked about EIR dissemination approaches and knowledge products, reported influence of EIR on policy and programme decisions, reported changes in service delivery, enabling and constraining factors, and continued application of EIR approaches. The tool was pilot-tested with UNICEF programme staff to ensure clarity and relevance.

### Data analysis

We analysed quantitative data descriptively to summarise the proportion of projects reporting specific outcomes. Qualitative data were analysed thematically using a combined inductive and deductive approach [15,16]. Deductive coding was guided by Proctor’s implementation outcomes and CFIR domains, while inductive coding facilitated the identification of emergent themes related to mechanisms, contextual influences, and the institutionalisation of EIR practices.

Two researchers independently reviewed and coded the data, iteratively refining the coding framework and resolving discrepancies through discussion. We organised results in the two analytical layers – implementation outcomes to outline the identified tangible policy and service delivery changes (adaptation, fidelity, sustainability) resulting from EIR findings and mechanisms and context to explore how and why these outcomes occurred, guided by the CFIR domains.

### Triangulation

To enhance the credibility of findings, we triangulated survey responses with multiple data sources, including project reports, dissemination materials, and UNICEF monitoring documentation. This triangulation enabled cross-validation of reported changes in programme, policy, and service delivery and provided additional context for interpreting the findings. Where evidence relies primarily on respondents' perspectives, results are presented as reported perceptions rather than independently verified outcomes.

## RESULTS

### Participation overview

Of the 48 eligible team members from 40 completed UNICEF-supported EIR projects, 33 respondents (21 researchers from universities or research institutions and 12 programme implementers) representing 24 EIR projects in 11 countries participated in the survey, yielding a 69% response rate (**Table 1**). Most EIR projects (79%) focused on the expanded programme on immunisation (EPI), reflecting a strong emphasis on addressing bottlenecks in immunisation service delivery. Other thematic areas for the EIR projects included prevention of mother-to-child transmission, birth registration, vitamin A supplementation, enhanced child health days, and broader maternal, newborn and child health (MNCH) programmes, capturing a diverse range of service delivery and systems challenges across contexts.

**Table 1:** Completed embedded implementation research projects supported by UNICEF, 2015–2022

Country	Thematic area	Research focus
Bangladesh	MNCH	Improving delivery of MNCH services in humanitarian (Rohingya) settings
Benin	Vitamin A supplementation	Increasing coverage of vitamin A supplementation and routine immunisation
Burkina Faso	Enhanced Child Health Days	Improving acceptability and adoption of routine vitamin A supplementation by CHWs and health workers
Chad	EPI	Improving access to immunisation services for hard-to-reach populations
Côte d’Ivoire	PMTCT	Strengthening communication strategies for pregnant adolescents and young mothers
Côte d’Ivoire	PMTCT	Understanding needs and preferences of teenage mothers for PMTCT services
Côte d’Ivoire	Enhanced Child Health Days	Using community platforms to improve vitamin A supplementation delivery
Democratic Republic of the Congo	EPI	Strengthening immunisation health information systems
Democratic Republic of the Congo	PMTCT	Increasing male partner involvement in PMTCT services
Ethiopia	EPI	Improving data use and accountability in immunisation programmes
Ethiopia	EPI	Strengthening caregiver tracking and feedback mechanisms across facilities
Ethiopia	Birth registration	Integrating civil registration into health systems
India	EPI	Addressing vaccine hesitancy linked to misinformation and social media
Kenya	EPI	Addressing vaccine hesitancy during new vaccine introduction
Madagascar	Vitamin A supplementation	Improving delivery of vitamin A supplementation services
Malawi	PMTCT	Assessing effectiveness of PMTCT programmes
Malawi	PMTCT	Integrating infant HIV testing into routine MNCH services
Malawi	Enhanced Child Health Days	Integrating vitamin A supplementation into routine immunisation services
Mali	Birth registration	Strengthening community health worker roles in civil registration
Nigeria	EPI	Improving immunisation coverage through participatory approaches
Nigeria	EPI	Enhancing demand for immunisation through civil society engagement
Nigeria	EPI	Addressing socio-contextual barriers to immunisation in urban slums
Pakistan	EPI	Improving immunisation coverage in urban slums
Pakistan	EPI	Addressing community-level barriers to immunisation uptake
Pakistan	EPI	Strengthening supportive supervision systems
Pakistan	EPI	Improving immunisation supply chain performance
Pakistan	EPI	Using mHealth to increase immunisation demand
Pakistan	EPI	Addressing vaccine hesitancy through social mobilisation
Pakistan	EPI	Strengthening referral systems through community health workers
Pakistan	EPI	Identifying barriers and enablers of digital immunisation systems (E-Vaccs)
Pakistan	EPI	Improving accountability in human resources for immunisation
Pakistan	EPI	Strengthening integration between EPI and polio programmes
Senegal	Birth registration	Strengthening interoperability in civil registration systems
Somalia	EPI	Strengthening local capacity for immunisation demand generation
South Sudan	Newborn in emergencies	Improving newborn care practices in conflict settings
South Sudan	Birth registration	Strengthening interoperability of community-based health and civil registration systems
Uganda	EPI	Strengthening leadership for integrated district health programmes
Uganda	EPI	Improving community-based microplanning for immunisation
Vietnam	EPI	Improving management of immunisation systems for children under two

CHW – community health workers, EPI – expanded programme on immunisation, MNCH – maternal, newborn and child health, PMTCT – prevention of mother-to-child transmission

### Impact on policy, programme and service delivery outcomes

**Figure 1:**
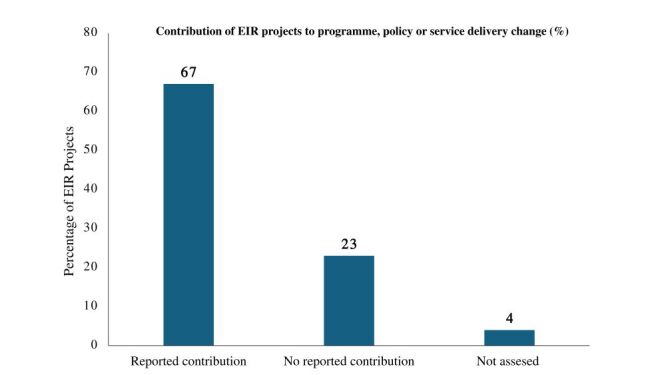
Contribution of UNICEF-supported embedded implementation research projects to service delivery, programme and policy changes, 2015–2022. Values represent the percentage of projects (n = 24) reporting contribution, no contribution, or where contribution was not assessed. EIR – embedded implementation research.

Regarding research findings, 16 of the 24 project teams (67%) reported that their research findings directly influenced policy or programmatic decisions (**Figure 1**). These included adaptations to immunisation strategies, supply-side service improvements, integration of digital technologies, and strengthened community engagement. EIR findings contributed to notable progress by introducing mHealth technology and recruiting new vaccinators, thereby enhancing supply-side service delivery at the provincial level in Pakistan. Malawi successfully scaled up vitamin A supplementation by integrating it into routine immunisation services nationwide. Important systems-level reforms were also documented in Uganda and Ethiopia. Uganda revised its immunisation microplanning tool to strengthen implementation of the EPI, while Ethiopia advanced birth registration by embedding it into MNCAH, EPI and community health platforms.

In Nigeria, communication and community mobilisation strategies were revised based on findings from the EIR. The improved communication strategies increased vaccine demand and contributed to improved immunisation coverage in two wards (**Table 2**). Similarly, the Chad team developed tailored communication and demand-generation approaches to reach nomadic populations through an integrated human-animal vaccination campaign. The team in India produced a health worker manual to strengthen vaccine literacy by addressing widespread misconceptions about immunisation (**Box 1**). This case study is included to illustrate how embedded implementation research can be applied to address programme challenges in a real-world setting. It complements the broader analysis by providing a concrete example of how evidence was generated and used to understand the drivers of vaccine hesitancy and inform context-specific strategies to improve immunisation uptake. Immunisation coverage increased from previously reported low levels (<72%) to 88.8% within the intervention area. This observation is based on programme-level data and was not formally evaluated using statistical methods; therefore, findings should be interpreted with caution.

**Table 2:** Reported programmatic, policy and service delivery changes resulting from embedded implementation research projects supported by UNICEF, 2015–2022

Country	Thematic area	Reported service delivery, programme or policy change
Nigeria	EPI	Improved community mobilisation and communication strategies in two wards, leading to increased immunisation coverage
Pakistan	EPI	Introduction of mHealth technology and recruitment of additional vaccinators to strengthen immunisation services
Pakistan	EPI	Federal government engagement in social media content management to reduce anti-vaccine misinformation
Pakistan	EPI	Improved supply-side service delivery through strengthened provincial health system actions
Kenya	EPI	Scale-up of pain management strategies across several hospitals to improve vaccination experience
Kenya	EPI	Improved provider knowledge and application of pain mitigation techniques to increase vaccination uptake
India	EPI	Development of a health worker manual to improve vaccine literacy and response to community misinformation
Chad	EPI	Enhanced communication strategies to reach nomadic populations through integrated human and animal vaccination campaigns
Uganda	EPI	Revision and improvement of the immunisation microplanning tool
Malawi	ECHD	National scale-up of Vitamin A supplementation through integration into routine immunisation services
Ethiopia	BR	Strengthened birth registration through integration into MNCH, EPI programmes, and community health platforms

BR – birth registration, EPI – expanded programme on immunisation, ECHD – enhanced child health days, MNCH – maternal, newborn and child health

Box 1 **Case study: tackling vaccine hesitancy in Malappuram, India [ 17]**

Research objective: to explore the root causes of vaccine hesitancy and mistrust in the immunisation programme in Malappuram district, Kerala, particularly in the context of anti-vaccine social media campaigns and to assess how trust deficits between caregivers and health workers impact vaccination decisions. Methods: a qualitative study was conducted through in-depth interviews and focus group discussions with a range of stakeholders, including parents/caregivers, physicians, public-sector health staff, traditional medicine practitioners, field healthcare workers, teachers, and communication experts. Research was focused on two areas of Malappuram with contrasting immunisation coverage. Key findings: vaccine hesitancy stemmed from a lack of trust between caregivers and health providers, fuelled by health workers' limited technical knowledge. Patriarchal societal norms and anti-vaccine messaging from naturopaths and homoeopaths. Social media played a central role in spreading anti-vaccine misinformation, and religion was not a significant factor in vaccine resistance in this context. Dissemination and advocacy strategy: findings were presented at the district-level workshop, the state-level workshop, two international Conferences, and at an in-person meeting with policymakers and the Health Minister. Moreover, the implementation research team, comprising researchers and the local immunisation programme manager, carried out continuous advocacy. Impact and outcomes: The implementation research team compiled a comprehensive list of anti-vaccine myths and arguments and developed a Malayalam-language compendium to equip field workers with scientifically sound responses. A mobile app was also created to help health workers counter misinformation in real-time. As a result, immunisation coverage at the Vattamkulam primary healthcare centre of Malappuram district (previously the lowest at <72%) rose to 88.8%, highlighting the effectiveness of the implementation research.

Half (50%) of projects reported positive effects on service delivery outcomes, particularly in immunisation coverage, outreach performance, and service quality. These improvements reflect enhanced fidelity in implementing evidence-informed practices and in integrating EIR findings into routine delivery processes. Among these, 85% attributed improvements to increased vaccination coverage, while others cited gains such as enhanced vitamin A supplementation coverage and improved community engagement strategies.

*Findings from the research led to improved routine immunisation coverage in the two wards in which the research was conducted. It has also improved our community engagement strategy.* – Implementer, EPI Nigeria

*[The] findings led to local leadership (cultural/religious) mobilising resources and running a campaign to promote improved immunisation to reach all children in the district. There has been improved networking, planning and coordination of immunisation activities.* – Implementer, EPI Nigeria

*IR results increased coverage of vitamin A supplementation in older children provided through routine [immunisation] services over time during implementation, following the strengthening of the weak health system. This also contributed to improving coverage of Measles-Rubella vaccine […]* – Implementer, MoH Benin

Evidence collected from relevant UNICEF offices triangulated these findings, showing measurable gains in service indicators after EIR-informed adaptations. Further, seven teams (29%) reported that their research findings had no impact on programmes or policies, often due to insufficient engagement with policymakers or delays in implementing the EIR project. Additionally, one team reported that they had not yet measured the impact of their IR findings or were unaware of any changes, sometimes due to disruptions such as the COVID-19 pandemic or ongoing processes.

*We have not assessed the outcome after changes in the implementation.* – University researcher, Pakistan

*Not yet. The change took more time than we expected. We are still working with district officials.* – University researcher, Ethiopia

### Mechanisms and contextual drivers influenced the implementation outcomes

#### Intervention characteristics: approach aligned with programme cycles and relevance to national priorities

We asked respondents about the key facilitators that contributed to service delivery, programme and policy changes. Several respondents (n = 11) emphasised that the EIR approach itself served as a major enabler, as it adopted a bottom-up design process in which research questions and study designs were co-developed with national health and immunisation programme managers and government decision-makers. This collaborative approach enhanced government ownership and ensured that the research directly addressed priority implementation bottlenecks. Respondents emphasised that EIR projects strategically aligned with existing programme planning and implementation cycles demonstrated substantially greater policy traction and facilitated more effective uptake of EIR findings into decision-making processes.

*Key enablers of policy uptake included early engagement with decision-makers, alignment with programme implementation cycles, and participatory dissemination and advocacy strategies.* – Implementer, EPI Nigeria

#### Inner setting: leadership engagement, effective coordination and institutional readiness

Respondents highlighted that EIR projects embedded within established coordination structures led by the Ministry of Health (MoH) benefited from strong leadership engagement and more consistent use of findings. Engaging programme implementers and decision-makers as co-principal investigators, alongside overall coordination by the MoH leadership, fostered trust and accelerated the translation of evidence into policy and practice.

*[The] findings led to local leadership (cultural/religious) mobilising resources and running a campaign to promote improved immunisation to reach all children in the district. There has been improved networking, planning and coordination of immunisation activities by the MoH.* – Implementer, EPI Pakistan

Respondents (n = 6) emphasised that EIR projects co-led by decision-makers and implemented in collaboration with competent research institutes with adequate capacity and strong working relationships with government counterparts catalysed policy and programme adaptation and contributed to measurable improvements in service delivery in LMICs.

*[…] It was easy to execute the findings faster, and access to policymakers was also easy, as the study was carried out by the Ministry of Health's staff and at their own facilities. It was easier to convince the policymakers with this kind or research rather than a third party doing the research and approaching the policymakers with the result […]* – University researcher, Chad

#### Outer setting: political commitment, policy environment and donor engagement

As highlighted by multiple respondents (n = 15), translating research into action was often constrained by the broader political, financial, and policy environment. Sustained commitment from the senior MoH leadership, active engagement of UNICEF and other development partners, including WHO, and establishment of effective follow-up mechanisms after dissemination of the EIR findings were identified as essential elements for maximising the outcomes and scalability of EIR across diverse contexts.

EIR teams from Pakistan and Ethiopia highlighted how strong external policy support, along with additional financing and technical contributions from the MoH and partners, to implement the EIR-derived recommendations substantially enhanced policy uptake and programme adaptation.

*Cross-country learning provided by the funders (UNICEF, WHO) was quite useful - not just for building networks in IR but for enabling refinement of approaches due to exposure to best practices.* – Implementer, EPI Pakistan

#### Characteristics of individuals: capacity strengthening and researcher -implementer co-ownership

UNICEF, in collaboration with research and development partners, organised capacity-building activities throughout the EIR process through research-priority setting, protocol development, and data analysis workshops. Respondents mentioned the value of being part of the EIR initiative, as it gave them the opportunity to build their EIR capacity. Notably, 87% (n/N = 29/33) of respondents reported applying skills and knowledge acquired through the EIR projects in subsequent professional activities, reflecting significant capacity gains and the potential for sustained impact beyond individual projects.

*So many skills were acquired relating to knowledge translation, stakeholder engagement, policy, etc. They have helped me to contribute to several other proposals and projects that I've been involved in since.* – Implementer, EPI Pakistan

*Even after the completion of the project, we continued using EIR methods in planning and supervision activities. It has now become part of how we work.* – Implementer, EPI India

The majority of the participants (80%) emphasised that EIR provided a structured mechanism for linking real-time evidence to programme decision-making, particularly when policymakers and frontline implementers collaborated with local researchers and research institutes were involved from the outset. The EIR model fostered close collaboration between implementers and researchers, enhancing team motivation and facilitating the timely completion of each project.

*I had the first-hand experience of knowing how the research findings are avidly taken up by the policy and system if they are involved right from the stage of developing study objectives and finalising its design.* – University researcher, Pakistan

### Process: participatory engagement for dissemination and continuous follow-up

All 24 project teams produced final project reports, and 67% (n/N = 16/24) published peer-reviewed articles. Policy briefs were developed by 62% of teams, often accompanied by implementation guides, videos and infographics to facilitate the use of findings and recommendations. Most teams (87%) produced multiple knowledge products, enhancing the reach and utility of their findings.

To promote policy and programme uptake, teams engaged in a range of dissemination activities to share their findings. All participated in multi-stakeholder workshops, while many also used complementary strategies: policymaker meetings (54%), roundtable discussions (29%), seminars (25%), and conference presentations (21%).

Nearly all teams (87%) employed more than one dissemination approach. Participatory dissemination was among the strongest mechanisms linking EIR results to decision-making, as highlighted by most of the respondents (n = 14). These activities facilitated engagement with stakeholders at multiple levels, from local implementers to national policymakers. Teams that developed several knowledge products, organised various dissemination strategies, and continuously followed up on the utilisation of the EIR findings with the relevant decision-makers reported faster adoption and inclusion of the results in policies and plans. Several respondents called for continued support to ensure sustained follow-up beyond project completion, noting that dissemination efforts alone do not guarantee meaningful impact.

*We have conducted a dissemination workshop gathering the policy makers at local and central level as well as the representatives of the communities.* – Implementer, ECHD programme Malawi

### Interaction between implementation outcomes and contextual facilitators

**Figure 2:**
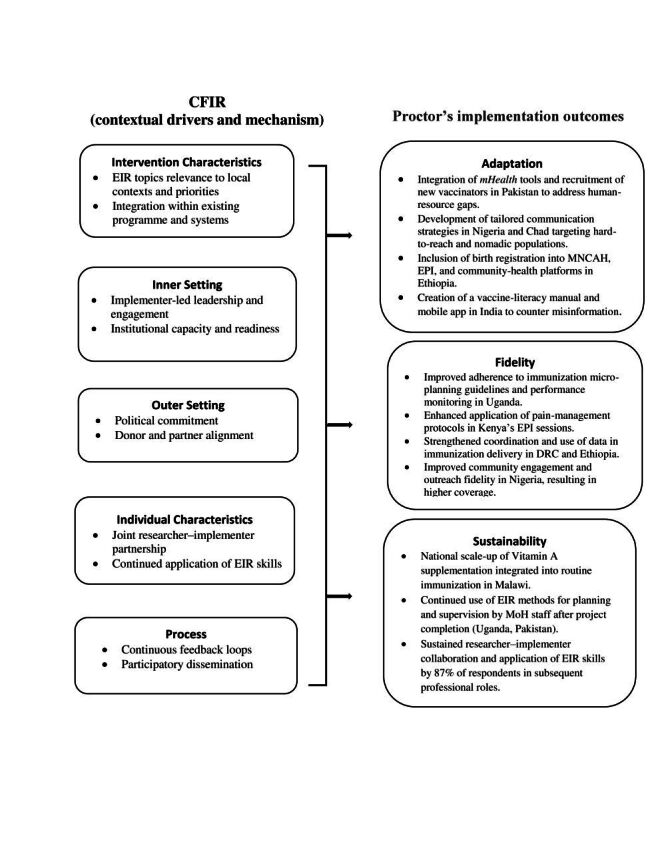
Interaction between CFIR domains and Proctor’s implementation outcomes illustrating how EIR findings influenced programme, policy and service delivery adaptation, fidelity, and sustainability across multiple LMIC contexts. CFIR – contextual drivers and mechanism, EIR – embedded implementation research, EPI – expanded programme on immunisation, LMIC – low- and middle-income country, MNCAH – Maternal, Newborn, Child and Adolescent Health.

To better understand how EIR led to observable changes across countries, we illustrated how contextual factors within the CFIR domains, such as leadership engagement, political commitment, and participatory dissemination, interacted with implementation outcomes (adaptation, fidelity, and sustainability) to shape policy, programme, and service-delivery improvements across multiple LMIC settings (**Figure 2**).

### Barriers to uptake of the EIR findings

Seven EIR teams indicated that their IR findings did not lead to policy, programmatic, or service-delivery changes. The most frequently cited obstacle was the lack of political commitment (reported by three teams) or limited interest from key decision-makers, even when those actors had been engaged during earlier stages of the research.

*The challenges were to get the attention of the decision-makers and convince them to propose the changes. [There was] not much attention given to the findings.* – University researcher, Pakistan

*Pushing the policy to the national level is still a challenge because of bureaucratic and logistical protocols.* – University researcher, Uganda

Additional barriers included insufficient funding for post-study implementation of the recommendations, competing priorities, leadership turnover, and challenges scaling interventions beyond pilot settings. In some instances, respondents noted missed opportunities for timely dialogue with policymakers, reducing the relevance or perceived urgency of the findings.

*There was no financial provision to integrate research findings at the district level.* –Implementer, EPI Pakistan.

## DISCUSSION

This multicountry synthesis demonstrates that EIR can contribute meaningfully to health policy adaptation, programme improvement, and, in many cases, enhanced service delivery outcomes. Embedded implementation research, when co-led by decision-makers and integrated into health systems, can catalyse policy and programme adaptation and contribute to measurable improvements in service delivery in LMICs. This analysis goes beyond established principles by identifying key mechanisms through which EIR operates, including co-leadership with decision-makers, alignment with programme cycles, and participatory dissemination. These findings, interpreted through CFIR and Proctor’s implementation outcomes framework, highlight how contextual and process-related factors interact to shape EIR outcomes.

Most surveyed EIR teams reported using multiple dissemination tools, including policy briefs, stakeholder workshops and peer-reviewed publications, which helped bring targeted attention to key stakeholders, supported sensitisation around programme challenges, and created pathways for engagement that facilitated uptake of EIR findings into decision-making [18–21]. These practices align with evidence showing that timely and targeted knowledge translation mechanisms are essential to promote evidence-informed decision-making and overcome implementation bottlenecks, enabling system responsiveness in LMIC settings [2,3,7].

Crucially, engagement of policymakers throughout the research process emerged as a consistent enabler of EIR uptake. Our findings reflect those of earlier analyses, indicating that co-leadership by implementers not only improves research relevance but also accelerates uptake and legitimacy of findings among local actors [10,11,22–25] and builds trust among implementers, decision-makers and other stakeholders with respect to the validity of the research findings and recommendations and in their relevance to the local context [2,3,7,24,25]. Further, compared to conventional research approaches, which often face delays between evidence generation and use, EIR allows faster translation of learning into programme adjustments [17,19,26,27]. This was evident in our analysis in Pakistan, India, and Malawi, where EIR directly shaped immunisation strategies and delivery platforms.

The study also highlights EIR’s role as a vehicle for capacity strengthening. Nearly 90% of the participants reported applying implementation research-acquired skills in other projects or academic work. This ripple effect suggests that EIR can contribute not only to programme performance but also to the broader, long-term capabilities of researchers, programme implementers, and institutions. In contexts where scientific and implementation research capacity is often underdeveloped, such gains are particularly valuable [21,28]. Importantly, these findings suggest not only enhanced individual competencies but also the continued application of EIR approaches within programmes, indicating the potential institutionalisation of evidence-informed practices beyond individual EIR projects.

Despite its considerable promise, the successful operationalisation of EIR remains constrained by several challenges. Across several country initiatives, teams reported limited institutional and political commitment to using implementation research findings, as well as constrained financial resources, competing programme priorities, and bureaucratic inertia. These barriers are widely recognised as limiting the translation of implementation research findings into policy, programme improvement, and sustained health systems changes [3,19,25,29–31].

Although EIR emphasises stakeholder involvement, our results suggest that meaningful, continuous engagement beyond initial buy-in remains difficult to maintain. Therefore, the practice of engaging with relevant policymakers at all stages, creating mutual trust, fostering continuous dialogue and advocacy are all critical for successful research uptake [2,3,19,29]. Moreover, ensuring that findings influence national-level policy rather than subnational pilot adaptation often requires additional advocacy, alignment of timing, and leadership continuity [30,32]. In addition, challenges relating to inadequate funding, sustainability and scalability of programme adaptations cited by the respondents have been noted in other studies on EIR [3,33].

Lastly, inadequate mechanisms for tracking outcomes of the EIR projects over time limited the ability of some teams to demonstrate changes in service delivery. Institutionalising outcome monitoring, even in small-scale implementation research, could enhance the credibility and policy utility of such work.

The study participants represented a broad and diverse range of EIR projects, approximately two-thirds of all UNICEF-supported IR projects, with perspectives primarily reflecting those of researchers and implementers. Although fewer implementers participated than researchers, the selected participants provided valuable insights into the practical application and impact of EIR across diverse contexts. This study was not designed to generate statistically representative estimates across all LMICs. Rather, it identifies recurring patterns and reported experiences across a diverse set of EIR projects. Findings should therefore be interpreted as indicative of common themes rather than generalisable to all settings. Future research incorporating policymakers’ perspectives, quantitative assessments of programme outcomes, and cost-effectiveness analyses of EIR-informed interventions would further strengthen understanding of the impact and value of EIR.

## CONCLUSIONS

This study reinforces the pivotal role of embedded implementation research in driving policy adaptation, programme refinement, and service delivery improvements in child health and other priority areas across LMICs. When embedded within existing coordination structures and co-led by decision-makers, EIR can transform the use of evidence from a reactive process into an institutionalised practice.

However, translating EIR findings into sustained action remains constrained by political, financial, and operational barriers. Strengthening political commitment, ensuring predictable financing, and establishing robust follow-up mechanisms are critical to sustaining momentum and scaling successful interventions.

Embedding capacity-building components within EIR initiatives adds enduring value by cultivating a culture of inquiry and equipping both researchers and programme managers to act on evidence. Realising the full potential of EIR requires moving beyond passive knowledge generation towards deliberate strategies that mobilise political will, align resources, and institutionalise evidence-informed policymaking within health systems.

## Data Availability

**Data availability:** The data supporting the findings of this study are available upon request from the corresponding author. The data are not publicly available due to privacy or ethical restrictions.
